# Variations of *CHI3L1*, Levels of the Encoded Glycoprotein YKL-40 and Prediction of Fatal and Non-fatal Ischemic Stroke

**DOI:** 10.1371/journal.pone.0043498

**Published:** 2012-08-24

**Authors:** Camilla Noelle Rathcke, Stine Brinkloev Thomsen, Allan Linneberg, Henrik Vestergaard

**Affiliations:** 1 Deptartment of Internal Medicine, Center of Endocrinology and Metabolism, Copenhagen University Hospital Herlev, Copenhagen, Denmark; 2 Department of Cardiology and Endocrinology, Copenhagen University Hospital Hilleroed, Copenhagen, Denmark; 3 Research Centre for Prevention and Health, Copenhagen University Hospital Glostrup, Copenhagen, Denmark; 4 Faculty of Health Sciences, University of Copenhagen, Copenhagen, Denmark; Leibniz-Institute for Arteriosclerosis Research at the University Muenster, Germany

## Abstract

**Background:**

Polymorphisms of *CHI3L1* are associated with inter-individual YKL-40 levels and YKL-40 is associated with an increased mortality and is elevated in patients with cardiovascular disease. We investigated the association between single nucleotide polymorphisms (SNPs) of *CHI3L1*, serum YKL-40 levels and all-cause and cardiovascular mortality and first-time incidence of myocardial infarction, ischemic heart disease (IHD) and stroke.

**Methodology/Principal Findings:**

12 SNPs of *CHI3L1* were genotyped and serum YKL-40 was measured in 2656 Danes representative of the general population. Median follow-up period was 15 (0–16) years. Admission data and deaths were ascertained from registers from the Danish National Board of Health. Fourth quartile YKL-40 levels were associated with an increased mortality risk of ischemic stroke (HR 2.44 (1.01–5.88), p = 0.041) and so were homozygotes of the minor allele of rs872129 (HR 9.35 (1.25–69.87, p = 0.022)). Both continuous YKL-40 levels and 4^th^ quartile YKL-40 values (>85 ng/ml) were associated with all-cause mortality (HRs 1.22 (95% CI, 1.10–1.35), p<0.0001, and 1.40 (1.15–1.71), p<0.0001), an increased risk of first-time stroke (HR 1.16 (1.01–1.33), p = 0.04, and 1.63 (1.23–2.16), p = 0.001) and a decreased risk of incidence of IHD (HR 0.77 (0.65–0.91), p = 0.002, and 0.61 (0.44–0.85), p = 0.003).

**Conclusions/Signficance:**

High YKL-40 levels (>85 ng/ml) and rs872129 were associated with an increased mortality risk of ischemic stroke, but high YKL-40 levels were also inverse related with the risk of incidence of IHD. This could be a chance finding but could also elucidate that YKL-40 plays different roles in development of thromboembolisms versus the formation of local thrombosis.

## Introduction

Substantial evidence indicates a pathogenic role of the inflammatory glycoprotein YKL-40 in endothelial dysfunction and the earliest part of the atherosclerotic process leading to disease progression and manifest cardiovascular disease (CVD) [1]. Several clinical studies document elevated YKL-40 levels in patients with CVD and an association between YKL-40 and mortality [1]. Cardiovascular studies show that elevated YKL-40 levels are associated with the presence [2]–[4] and extent [2] of coronary artery disease (CAD), indicating that YKL-40 levels could be a quantitative indicator of disease presence and progression [2]. Elevated YKL-40 levels are documented in patients having acute myocardial infarction (MI) [3]–[5], and are also associated with all-cause and cardiovascular mortality in patients with stable CAD [4] and in individuals representative of the general population [6], [7]. Recently, elevated YKL-40 levels have been found associated with an increased risk of ischemic stroke [8].

YKL-40 seems especially involved in activation of the innate immune system and is secreted by a variety of cells [9]. YKL-40 mRNA expression is highly up-regulated in distinct subsets of macrophages in the atherosclerotic plaque [10]. Particularly macrophages that had infiltrated deeper in the lesion show high YKL-40 mRNA expression and the highest expression is seen in macrophages in the early lesion of atherosclerosis [10]. YKL-40 is encoded by the chitinase 3-like1 gene, *CHI3L1*, and several studies of single nucleotide polymorphisms (SNPs) of *CHI3L1* show that genetic variations of *CHI3L1* have an impact on inter-individual serum YKL-40 levels and asthma susceptibility [11]–[14]. Only a single association study of polymorphisms of the *CHI3L1* locus and cardiovascular disease have been conducted [15]. It has been documented that the polymorphisms rs10399931 and rs4950928 were associated with YKL-40 levels, but not with prevalence or severity of CAD [15].

The objectives of the present study were to investigate 1) the putative association of common variations in the *CHI3L1* locus with inter-individual serum YKL-40 levels and the prevalent odds ratio of MI and stroke, and 2) the putative association of SNPs and circulating YKL-40 levels with all-cause and cardiovascular mortality and with first-time incidence of MI, IHD and stroke over a 15 years period in 2656 individuals representative of the general population.

## Methods

### Ethics Statement

All participants gave informed written consent to participation in both the initial and the derivative study, which both were approved by the local Ethical Committee of Copenhagen County and conducted in accordance with the Helsinki Declaration.

### Study Design

In 1982 an age- and gender stratified sample consisting of 4807 men and women, born in 1922, 1932, 1942 and 1952 (aged exactly 30, 40, 50 and 60 years), residing in the western part of Copenhagen County, was drawn from the National Danish Civil Register in which all people living in Denmark are registered by a unique 10-digit number. The sample size was reduced to 4581 Danes because of the exclusion of 226 individuals of foreign origin. Socio-demographic factors in the sampling area were compared with national statistics to ensure sample validity [16]. All sample members were invited to a general health examination and to complete a questionnaire concerning lifestyle, health and medical history [16]. Between November 1982 and February 1984, 3608 individuals (78.8%) entered the study. All participants underwent a general medical examination and blood samples were drawn for the studies planned at that time [17]–[19].

In 1993-94, cardiovascular studies comprising more specific cardiovascular examinations as e.g. echocardiography and aortic pulse wave velocity were initiated. All 3608 former participants were re-invited, and 2656 (73.6%) individuals, now of age 41–73 years, accepted and participated in new and further clinical examinations including measurements of height, weight and blood pressure. The questionnaire regarding lifestyle, health and medical history was updated and new blood samples were drawn.

### Clinical Examinations and Biochemical Measurements

A trained nurse retrieved anthropometric measures, and waist-to-hip ratio was calculated on the basis of the widest circumferences between the lower rib and the iliac crest and around the hips at the level of the major femoral trochanters. A standard electrocardiogram was performed and following 5 minutes of rest, arterial blood pressure was measured twice in the sitting position with the arm of the participant at the sternum level using a random zero mercury sphygmomanometer. Mean blood pressure was calculated. Heart rate was counted over 15 s and calculated per minute. Blood samples were obtained in the fasting state for standard analyses including glucose and parameters of the lipid profile, analyses of inflammatory and cardiac markers and for genotyping of *CHI3L1* polymorphisms.

Serum YKL-40 was determined with a commercial ELISA assay (Quidel, USA), measuring range 20 to 300 ng/ml. Serum high-sensitive C-reactive protein (hsCRP) was determined using a particle-enhanced immunoturbidimetric assay (Roche/Hitachi), measuring range 0.1–20 mg/l. Serum N-terminal fragment of the prohormone brain natriuretic peptide (NT-proBNP) was determined using Elecsys proBNP sandwich immunoassay on a Elecsys 2010 (Roche Diagnostics). Urine albumin concentration was determined by standard methods [20] using a turbidimetric method (Hitachi 717 analyzer, Roche Diagnostics) on a single morning urine specimen. Urine creatinine was assessed by the Jaffé reaction without deproteinizing and quantified by a photometric method (Hitachi 717 analyzer, Roche Diagnostics). Biomarker analyses success rate was >99.4% (N = 2642).

### Genotyping of Single Nucleotide Polymorphisms in the CHI3L1 Gene

A region 22 kb upstream and 10 kb downstream of *CHI3L1* were chosen from the HapMap project (www.hapmap.org) and HapMap Data Rel 21a/phaseII Jan07, on NCBI assembly, dbSNP b125, were used for the SNP selection. A total of 12 SNPs located in the region 14 kb upstream to 2 kb downstream of *CHI3L1* and covering all linkage disequilibrium (LD) blocks in *CHI3L1* were genotyped. TAGGER [21] chose these SNPs as the most informative in the chosen +22 kb−−10 kb region. TAGGER was used with a 5% minor allele frequency (MAF) cut off and aggressive tagging, i.e. r^2^>0.8. Genotyping was performed using KBiosciences allele-specific PCR (KASPar) (Kbioscience, Herts, UK) with a success rate >96.2%, which is equivalent to N = 2554 (variation of N = 2527–2571 between the different SNPs). Genotype distribution obeyed Hardy Weinberg equilibrium (HWE), all p>0.14 using Genepop v4.0.10 [22], [23].

### Outcomes

In October 2009, admission data of the participants in the follow-up period, which have been validated and described thoroughly previously [24], were extracted from central discharge registers from the Danish National Board of Health specified on codes of diagnoses from the International Classification of Diseases (ICD), 10^th^ revision. Similarly, deaths since study start were ascertained from central registers from the Danish National Board of Health, confirmed by the Danish Civil Personal Register, which records all deaths in Denmark, and cross-checked on blinded classification of death certificates. Overall, participants were followed for a median period of 15 years (range 0–16 years).

Primary endpoints in accordance to YKL-40 levels and *CHI3L1* polymorphisms were all-cause mortality and mortality from IHD (ICD-10 code I20-25.9), heart failure (HF) (I50-50.9), hemorrhagic stroke (I61-62) and ischemic stroke (I63-64). Cancer mortality (C00–C97) was evaluated to explore the possible association between YKL-40 levels and all-cause mortality. All ICD-10 codes were specified individually. Secondary non-fatal cardiovascular endpoints were defined as first-time incidence of MI (I21-22.9), IHD (I20-25.9) or stroke (I61-64) during follow-up.

### Definitions

Hypertension was defined as a systolic blood pressure ≥140 mm Hg, a diastolic blood pressure ≥90 mm Hg or use of antihypertensive drugs. Hypercholesterolemia was defined as use of cholesterol lowering drugs or a baseline serum cholesterol level >5 mmol/l. Low HDL was defined as serum HDL<1.0 mmol/l (male) or <1.2 mmol/l (female). Baseline MI and baseline stroke were defined as self-reported or central registered prior diagnoses of MI (I21-22.9) or stroke (I61-64), respectively. Baseline IHD was defined on the basis of angina London Score [25], effective use of nitro spray or a central registered prior diagnosis of IHD (I20-I25.9). Baseline diabetes was defined as a self-reported or registered prior diagnosis of diabetes (E10-15), ongoing treatment with antidiabetic agents or a baseline fasting plasma glucose level >6.9 mmol/l. First-time incidences of any of the events were defined as a diagnosis registered during follow-up.

### Statistical Analyses

Analyses were made with the statistical software package SPSS 18.0 (SPSS inc., Chicago, IL). P-values were two-sided, and p-values<0.05 were considered statistically significant. Study population were described according to quartiles of YKL-40 and 14 participants were excluded from statistical analyses due to missing YKL-40 analyses. Categorical data were compared with the chi-square test for k independent samples. Continuous data were compared with One-Way ANOVA. Nonparametric testing of distributions was made with Kruskal-Wallis test. Data with a non-Gaussian distribution was logarithmically transformed using the natural logarithm. Analyses of intercorrelations and correlates of YKL-40 were performed using univariate linear regression analyses. Based on univariate analyses, a 3-step backward multivariate regression analysis based on the R values of individual correlates were made using age and gender followed by adjustment for strong (R>0.20) and less strong (R = 0.11–0.20) individual predictors. Associations between *CHI3L1* polymorphisms and YKL-40 levels were examined in linear regression models with correlations between major allele homozygosity for the individual SNP and circulating YKL-40 levels reported as β coefficients with 95% confidence intervals (95% CI). Associations between *CHI3L1* polymorphisms and baseline prevalence of dichotomous outcomes were examined by logistic regression analyses and reported as odds ratios (ORs) with 95% CI. Differences were tested by the likelihood ratio test. Backward conditional Cox regression analyses based on time from inclusion to end of follow-up were used to assess adjusted hazard ratios (HRs) and 95% CI for each SNP and for continuous and 4^th^ quartile levels with each outcome. Hazard ratios for a doubling of continuous serum YKL-40 were assessed by calculating HRs of an increase in 1 U of base 2 logarithm of YKL-40 levels. Individuals with prior self reported events or ICD-10 diagnosed registered events of the specific analysed outcome were excluded from first-time incidence analyses.

## Results

Median serum YKL-40 level in the total study population was 57 ng/ml, interquartile range (IQR) 40–85 ng/ml. Characteristics at baseline according to YKL-40 quartiles are presented in Table 1. The highest YKL-40 levels were seen in male and older individuals and among smokers (p<0.0001). Individuals in the two highest YKL-40 quartiles had a higher prevalence of diabetes (p<0.0001), angina pectoris (p<0.001) and previous cases of MI (p<0.002), and for the highest YKL-40 quartile alone also a higher prevalence of claudicatio intermittens (p<0.0001). Higher systolic and diastolic blood pressure and a higher prevalence of hypertension were seen among individuals with the highest YKL-40 levels (all p<0.0001). In the upper two YKL-40 quartiles, individuals also presented with a higher BMI, WHR, plasma glucose level, a more pronounced insulin resistance and a higher prevalence of diabetes (all p<0.0001). Whereas total cholesterol levels and prevalence of hypercholesterolemia increased (p<0.0001), neither did LDL increase (p = 0.06) nor HDL decrease (p = 0.63) with increasing YKL-40 levels. However, triglyceride levels and the prevalence of individuals with low HDL was higher among individuals in the highest YKL-40 quartile (p<0.0001). Finally, increasing levels of hsCRP and NT-proBNP were seen with increasing YKL-40 quartiles (all p<0.0001).

**Table 1:** Characteristics of the study population at baseline grouped according to quartiles of serum YKL-40.

	1^st^ quartile	2^nd^ quartile	3^rd^ quartile	4^th^ quartile	p value
YKL-40 range, ng/ml	≤40	>40, ≤57	>57, ≤85	>85	
N	677	672	633	660	<0.0001
Male	302 (44.6)	327 (48.7)	316 (49.9)	378 (57.3)	<0.0001
41–43 years	266 (39.3)	224 (33.3)	142 (22.4)	91 (13.8)	<0.0001
51–53 years	226 (33.4)	201 (29.9)	169 (26.7)	143 (21.7)	<0.0001
61–63 years	134 (19.8)	165 (24.6)	166 (26.2)	211 (32.0)	<0.0001
71–73 years	51 (7.5)	82 (12.2)	156 (24.6)	215 (32.6)	<0.0001
Smoking*	229 (33.8)	265 (39.4)	299 (47.2)	318 (48.2)	<0.0001
Alcohol consumption	576 (85.7)	571 (85.4)	532 (85.4)	586 (89.2)	<0.0001
Angina pectoris	21 (3.1)	25 (3.7)	40 (6.3)	49 (7.4)	<0.001
Claudicatio	3 (0.4)	1 (0.1)	2 (0.3)	5 (0.8)	<0.0001
Previous MI	14 (2.1)	11 (1.6)	26 (4.1)	31 (4.7)	0.002
Previous stroke	5 (0.7)	8 (1.2)	6 (0.9)	10 (1.5)	0.56
BP lowering medicine	64 (9.5)	52 (7.7)	101 (16.0)	124 (18.8)	<0.0001
BMI, kg/m^2^ †	25.2 (3.7)	25.8 (3.9)	26.2 (4.3)	26.7 (4.6)	<0.0001
WHR†	0.86 (0.09)	0.87 (0.08)	0.89 (0.08)	0.90 (0.09)	<0.0001
Systolic BP, mm Hg†	125 (17)	126 (18)	131 (20)	136 (19)	<0.0001
Diastolic BP, mm Hg†	80 (10)	81 (10)	83 (11)	84 (11)	<0.0001
Puls, bpm†	65 (10)	64 (10)	66 (11)	69 (12)	<0.0001
Hypertension	220 (32.5)	225 (33.5)	275 (43.4)	352 (53.3)	<0.0001
Glucose, mmol/l†	4.7 (0.6)	4.8 (0.9)	5.0 (1.3)	5.2 (1.4)	<0.0001
HOMA-IR	1.00 (0.92–1.10)	1.06 (1.00–1.12)	1.29 (1.18–1.40)	1.71 (1.55–1.86)	<0.0001
Diabetes	11 (1.6)	8 (1.2)	17 (2.7)	42 (6.4)	<0.0001
UACR, mg/g†	2.2 (1.0–4.0)	2.1 (0.8–4.1)	2.4 (0.9–4.7)	2.5 (0.8–7.4)	0.002
Cholesterol, mmol/l†	6.0 (1.0)	6.1 (1.1)	6.2 (1.1)	6.4 (1.1)	<0.0001
Hypercholesterolemia	311 (45.9)	340 (50.6)	345 (54.6)	413 (62.6)	<0.0001
LDL, mmol/l†	3.9 (1.0)	4.0 (1.0)	4.1 (1.0)	4.1 (1.0)	0.06
HDL, mmol/l†	1.5 (0.4)	1.4 (0.4)	1.4 (0.4)	1.5 (0.5)	0.63
Low HDL†	59 (8.7)	82 (12.2)	81 (12.8)	92 (13.9)	0.021
Triglycerides, mmol/l†	1.2 (0.8)	1.3 (0.8)	1.5 (0.9)	1.9 (1.4)	<0.0001
CRP, mg/l‡	1.2 (0.6–2.5)	1.4 (0.7–3.1)	2.2 (1.0–4.4)	2.8 (1.4–5.9)	<0.0001
NT-proBNP, pg/ml‡	39.8 (19.3–75.1)	47.9 (21.4–84.5)	59.2 (24.7–112.9)	63.7 (28.0–138.7)	<0.0001

* Smoking was defined as persons smoking one or more cigarettes/cigars/pipes a day; all others were classified as non-smokers.

Values are presented as.

† mean (SD),

‡ median (IQR) or N (% within quartile) where not specified.

Abbreviations not mentioned in main text: BP, blood pressure; HOMA-IR, homeostasis model assessment of insulin resistance.

### Correlates of YKL-40

The strongest correlates of YKL-40 levels were triglyceride level (R = 0.29), age (R = 0.28), hsCRP (R = 0.26), WHR (R = 0.24), systolic blood pressure (R = 0.23) and alcohol consumption (R = 0.21), all p<0.0001. Less strong correlates were pulse (R = 0.20), plasma glucose and insulin resistance (both R = 0.19), hypertension (R = 0.18), NT-proBNP (R = 0.15), smoking, total cholesterol and diabetes (all R = 0.12), BMI, diastolic blood pressure and male gender (all R = 0.11), all p<0.0001. Urine albumin/creatinine-ratio (UACR) only weakly determined YKL-40 levels (R = 0.08, p<0.0001).

Age and gender explained 30% (p<0.0001) of YKL-40 levels increasing to 48% (p<0.0001) when combined with WHR, alcohol consumption, triglyceride and hsCRP levels. Systolic blood pressure was excluded during backward regression (β = 0.03, p = 0.18). When also including less strong correlates (pulse, insulin resistance, plasma glucose, hypertension, NT-proBNP, smoking, total cholesterol, diabetes, BMI and diastolic blood pressure), 52% of the YKL-40 level could be explained (p<0.0001).

### Single nucleotide polymorphisms of CHI3L1

Prevalence of the 12 SNPs of *CHI3L1*, associated observed YKL-40 level for each genotype and correlations with circulating YKL-40 levels are shown in Table 2. All SNPs presented MAFs>5% except for rs4950930 (MAF = 4.7%). Rs4950930 was also the only SNP not to present significant differences in YKL-40 levels between genotypes. Five SNPs (rs10399931, rs2486064, rs4950928, rs880633 and rs946263) were positively correlated with circulating YKL-40 levels, especially rs10399931, rs4950928 and rs946263 presented strong correlations (R^2^ = 0.38, R^2^ = 0.40 and R^2^ = 0.38, all p<0.0001). The remaining 7 SNPs presented weak negative correlations with YKL-40 levels. None of the genotypes of any of the SNPs showed significant associations with prevalence of either MI (all p>0.22) or stroke (all p>0.09) (Table S1).

**Table 2:** Prevalence of the 12 single nucleotide polymorphisms (SNPs) of *CHI3L1*, associated observed serum YKL-40 levels for each genotype and association with serum YKL-40 levels.

SNP	Genotype distribution		YKL-40, ng/ml		Association with YKL-40 levels**	
	All	Prevalence, N (%)	Median (IQR)	p value	R^2^, β (95% CI)	p value
rs10399931	CC*	1478 (57.9)	66 (49–97)	<0.0001	R^2^ = 0.38	<0.0001
	CT	933 (36.6)	47 (35–68)		β = 0.44 (0.38; 0.49)	
	TT	141 (5.5)	27 (20–40)			
rs12123883	TT*	2165 (84.3)	56 (40–84)	0.013	R^2^ = 0.23	0.014
	TC	391 (15.2)	58 (43–86)		β = −0.07 (−0.13; −0.02)	
	CC	13 (0.5)	84 (55–233)			
rs2486064	GG*	811 (31.8)	65 (48–97)	<0.0001	R^2^ = 0.28	<0.0001
	GA	1247 (49.0)	55 (39–84)		β = 0.21 (0.18; 0.25)	
	AA	490 (19.2)	44 (31–67)			
rs2886117	GG*	1944 (76.9)	54 (38–80)	<0.0001	R^2^ = 0.24	<0.0001
	GA	542 (21.5)	65 (47–99)		β = −0.19 (−0.24; −0.14)	
	AA	41 (1.6)	72 (51–120)			
rs4950928	CC*	1592 (62.2)	66 (49–97)	<0.0001	R^2^ = 0.40	<0.0001
	CG	854 (33.4)	45 (34–64)		β = 0.45 (0.42; 0.49)	
	GG	113 (4.4)	26 (19–35)			
rs4950930	GG*	2319 (90.9)	56 (40–84)	0.051	R^2^ = 0.22	0.004
	GA	225 (8.8)	65 (45–93)		β = −0.11 (−0.18; −0.04)	
	AA	7 (0.3)	56 (46–84)			
rs6691378	GG*	2001 (78.4)	54 (39–80)	<0.0001	R^2^ = 0.24	<0.0001
	GA	518 (20.3)	66 (47–100)		β = −0.20 (−0.25; −0.15)	
	AA	33 (1.3)	81 (53–120)			
rs871799	GG*	2076 (81.2)	55 (39–82)	<0.0001	R^2^ = 0.24	<0.0001
	GC	448 (17.5)	64 (45–96)		β = −0.14 (−0.20; −0.09)	
	CC	32 (1.3)	67 (49–110)			
rs872129	AA*	2160 (84.4)	55 (39–82)	<0.0001	R^2^ = 0.23	<0.0001
	AG	386 (15.1)	64 (45–100)		β = −0.15 (−0.21; −0.09)	
	GG	12 (0.5)	60 (47–112)			
rs880633	CC*	734 (28.7)	62 (47–89)	<0.0001	R^2^ = 0.25	<0.0001
	CT	1284 (50.3)	56 (40–86)		β = 0.15 (0.12; 0.18)	
	TT	536 (21.0)	49 (32–75)			
rs883125	CC*	1842 (71.6)	53 (38–80)	<0.0001	R^2^ = 0.24	<0.0001
	CG	673 (26.2)	64 (45–98)		β = −0.15 (−0.19; −0.10)	
	GG	56 (2.2)	63 (49–86)			
rs946263	AA*	1647 (64.6)	65 (47–97)	<0.0001	R^2^ = 0.38	<0.0001
	AG	812 (31.8)	45 (33–64)		β = 0.44 (0.41; 0.48)	
	GG	92 (3.6)	26 (19–36)			

* Major allele.

** Adjusted for age, gender, triglycerides, alcohol, CRP and WHR.

### Primary endpoints

A total of 470 (17.8%) deaths occurred in the study population during follow-up (Table 3). Of these, 133 (28.3%) deaths were caused by cardiovascular disease (ischemic or hemorrhagic stroke, IHD or HF). All-cause mortality and mortality from ischemic or hemorrhagic stroke, IHD or HF during follow-up increased with increasing YKL-40 quartiles (Table S2). Both continuous and 4th quartile YKL-40 values were predictive of all-cause mortality with HRs of 1.23 (95% CI, 1.11–1.36) and 1.40 (95% CI, 1.15–1.71), p<0.0001, and 4th quartile YKL-40 values were also predictive of mortality of ischemic stroke after multivariate adjustement (HR 2.44 (95% CI, 1.03–5.88), p = 0.041) (Table 3). For individuals suffering a fatal ischemic stroke, there was no difference in age (p = 0.55), systolic blood pressure (p = 0.65), smoking status (p = 0.07) or alcohol consumption (p = 0.52) across YKL-40 quartiles. Neither continuous nor 4th quartile YKL-40 values were predictive of mortality of ischemic or hemorrhagic stroke, IHD or HF (Table S2).

**Table 3:** Hazard risks (HR (95% confidence interval) of all-cause mortality and mortality from ischemic heart disease (IHD), ischemic and hemorrhagic stroke and heart failure as predicted by continuous YKL-40 levels and 4^th^ quartile YKL-40 values.

	Continuous YKL-40 levels		4th quartile YKL-40 levels	
	All-cause	p value	All-cause	p value
Events, N	470		470	
Unadjusted	1.75 (1.62–1.89)	<0.0001	2.81 (2.34–3.36)	<0.0001
Adjusted for age and gender	1.40 (1.29–1.54)	<0.0001	1.61 (1.33–1.94)	<0.0001
Multivariable adjustement	1.23 (1.11–1.36)*	<0.0001	1.41 (1.15–1.72)*	0.001

	Ischemic stroke	p value	Ischemic stroke	p value
Events, N	22		22	
Unadjusted	1.92 (1.38–2.69)	<0.0001	4.90 (2.10–11.47)	0.01
Adjusted for age and gender	1.55 (1.03–2.34)	0.036	2.49 (1.05–5.92)	0.038
Multivariable adjustement	1.44 (0.92–2.28)†	0.11	2.44 (1.01–5.88)†	0.041

	Hemorrhagic stroke	p value	Hemorrhagic stroke	p value
Events, N	18		18	
Unadjusted	1.72 (1.32–2.24)	<0.0001	2.50 (1.34–4.69)	0.004
Adjusted for age and gender	1.25 (0.90–1.74)	0.18	1.18 (0.62–2.25)	0.61
Multivariable adjustement	1.07 (0.75–1.54)†	0.713	0.92 (0.47–1.83)†	0.82

	IHD	p value	IHD	p value
Events, N	79		79	
Unadjusted	1.67 (1.38–2.03)	<0.0001	2.82 (1.81–4.40)	<0.0001
Adjusted for age and gender	1.23 (0.98–1.54)	0.077	1.44 (0.91–2.28)	0.12
Multivariable adjustement	1.11 (0.87–1.42)‡	0.411	1.27 (0.79–2.05)‡	0.328

	Heart failure	p value	Heart failure	p value
Events, N	14		14	
Unadjusted	2.29 (1.57–3.34)	<0.0001	6.24 (2.09–18.63)	0.001
Adjusted for age and gender	2.01 (1.27–3.19)	0.003	3.19 (1.05–9.68)	0.04
Multivariable adjustement	1.54 (0.88–2.70)§	0.133	2.53 (0.80–8.01)§	0.114

* Adjustments: age, gender, smoking, alcohol, WHR, hypertension, baseline diabetes, baseline stroke, baseline IHD, total cholesterol, CRP, NT-proBNP, UACR.

† Adjustments: age, gender, smoking, hypertension, baseline diabetes, baseline stroke, baseline IHD, total cholesterol, HDL, CRP, NT-proBNP, UACR.

‡ Adjustments: age, gender, smoking, WHR, hypertension, baseline diabetes, baseline IHD, total cholesterol, HDL, CRP, NT-proBNP, UACR.

§ Adjustments: age, gender, smoking, hypertension, baseline diabetes, baseline IHD, total cholesterol, CRP, NT-proBNP, UACR.

Minor allele homozygosity of rs872129 was predictive of mortality of ischemic stroke with a HR of 9.35 (95% CI, 1.25–69.87), p = 0.029. Minor allele homozygosity of rs872129 only occurred in 0.5% of the total study population (N = 12, Table 2), but among the 22 individuals suffering a fatal ischemic stroke, the occurrence of minor allele homozygosity of rs872129 was 13.6% (N = 3, p<0.0001). None of the other SNPS were predictive of an increased risk of any of the primary endpoints (data not shown). Cancer was the cause of 177 (37.7%) deaths and both continuous and 4th quartile YKL-40 values were predictive of mortality from cancer with HRs of 1.19 (95% CI, 1.01–1.41), p = 0.039 and 1.46 (95% CI, 1.01–2.02), p = 0.024.

### Secondary endpoints

Incidences of MI and stroke, but not of IHD in general, increased with increasing YKL-40 quartiles (Table S2). Incidence of MI increased from 3.5% and 3.4% in the 1^st^ and 2^nd^ YKL-40 quartile to 4.3% in the 3^rd^ quartile and 5.0% in the 4^th^ quartile, p<0.0001. Similar, from the 1^st^ to the 4^th^ quartile incidences of stroke were 4.4%, 7.3%, 8.1% and 14.5% (p<0.0001). Both continuous and 4th quartile YKL-40 values were predictive of an increased risk of stroke (HRs 1.16 (95% CI, 1.01–1.33), p = 0.004, and 1.63 (95% CI, 1.23–2.16), p = 0.001) but at the same time also seemed protective against IHD in general (HRs 0.77 (95% CI, 0.65–0.91), p = 0.002, and 0.61 (95% CI, 0.44–0.85), p = 0.003) (Table 4). None of the levels were predictive of MI. Similar analyses of the SNPs showed that none of the genotypes of any of the SNPs were predictive of first-time events of MI, IHD or stroke (data not shown).

**Table 4:** Hazard risks (HR (95% confidence interval), 1 SD increase in ln2 variable) of first-time incidence of myocardial infarction, ischemic heart disease (IHD) and stroke during follow-up as predicted by continuous YKL-40 levels and 4^th^ quartile YKL-40 values.

	Continuous YKL-40 levels		4th quartile YKL-40 levels	
	Myocardial infarction	p value	Myocardial infarction	p value
Events, N	103		103	
Unadjusted	1.12 (0.92–1.37)	0.25	1.31 (0.86–1.99)	0.21
Adjusted for age and gender	0.92 (0.74–1.14)	0.44	0.92 (0.59–1.42)	0.69
Multivariable adjustement	0.82 (0.64–1.04)*	0.103	0.73 (0.46–1.17)*	0.195

	Stroke	p value	Stroke	p value
Events, N	226		226	
Unadjusted	1.43 (1.27–1.61)	<0.0001	2.36 (1.82–3.08)	<0.0001
Adjusted for age and gender	1.22 (1.07–1.39)	0.003	1.65 (1.26–2.16)	<0.0001
Multivariable adjustement	1.16 (1.01–1.33)†	0.041	1.63 (1.23–2.16)†	0.001

	IHD	p value	IHD	p value
Events, N	231		231	
Unadjusted	1.04 (0.91–1.20)	0.55	0.99 (0.73–1.33)	0.92
Adjusted for age and gender	0.88 (0.76–1.02)	0.10	0.73 (0.54–1.00)	0.05
Multivariable adjustement	0.77 (0.65–0.91)‡	0.002	0.61 (0.44–0.85)‡	0.003

* Adjustments: age, gender, smoking, WHR, hypertension, baseline diabetes, baseline angina pectoris, total cholesterol, HDL, CRP, NT-proBNP, UACR.

† Adjustments: age, gender, smoking, hypertension, atrial fibrillation, baseline diabetes, baseline IHD, total cholesterol, HDL, CRP, NT-proBNP, UACR.

‡ Adjustments: age, gender, smoking, WHR, hypertension, baseline diabetes, total cholesterol, HDL, CRP, NT-proBNP, UACR.

## Discussion

This study is the first to investigate the associations between genetic variants of the YKL-40-encoding gene *CHI3L1*, circulating YKL-40 levels and all-cause and cardiovascular mortality and development of first-time cardiovascular events in the general population. We documented, that 4^th^ quartile YKL-40 values (>85 ng/ml) were associated with an approximately 2.5 increased mortality risk of ischemic stroke over a time period of 15 years. This association could be attributable to the SNP rs872129 which were associated with a more than 9 times increased mortality risk of ischemic stroke and minor allele homozygosity of rs872129 was over represented among individuals suffering a fatal ischemic stroke. However, since only 0.5% of the study population was minor allele homozygotes of rs872129 this could be a chance finding and final conclusions cannot be made. Moreover, we also documented that a doubling in serum YKL-40 and 4th quartile YKL-40 values were associated with a 16% respectively 63% increased risk of first-time stroke, which are in accordance with findings in the Copenhagen City Heart Study [8]. Although the YKL-values in the present study were not categorized as in the Copenhagen City Heart Study, the risks of ischemic stroke are within the same range in both studies [8].

We also found that a doubling in serum YKL-40 and 4^th^ quartile YKL-40 values were associated with a 22% respectively 40% increased risk of all-cause mortality. This could most likely be attributable to the association between YKL-40 and cancer mortality although an association between YKL-40 levels and all-cause mortality in individuals representative of the general population has been documented previously in studies of both shorter duration of follow-up (5-6 years) [6], [26] and long term studies (16 years) [7] even when corrected for the influence of cancer [6]. The association between YKL-40 and all-cause mortality has also been documented in patients with stable CAD [4].

Neither a doubling in serum YKL-40 nor 4^th^ quartile YKL-40 values were associated with mortality from IHD, hemorrhagic stroke or heart failure. This is contradictory to a previous study showing an association between YKL-40 levels and cardiovascular mortality in general in individuals aged 50–89 years [6]. Although the analyses in both studies are age-adjusted, differences in age of the participants between the studies cannot be ruled out as a possible explanation. Moreover, the previous analyses were not adjusted for WHR and alcohol consumption, both correlates of YKL-40 levels in the present study. A previous study with a larger number of cardiovascular deaths documents an association between increasing YKL-40 levels and ischemic cardiovascular mortality [7]. However, the association was described differently (YKL-40 percentile category by gender and 10-year age group), and “ischemic cardiovascular events” were not defined making clarification of differences unachievable. Furthermore, in contrast to the present study, no adjustments for NT-proBNP levels were done.

Neither a doubling in serum YKL-40 nor 4th quartile YKL-40 values were associated with an increased risk of first-time MI. This is also in accordance with findings in the Copenhagen City Heart Study [8]. However, in the CLARICOR trial, YKL-40 predicted cardiovascular mortality, but no adjustments for other cardiac or inflammatory markers were made, and like in the present study, YKL-40 was not predictable of MI [4]. Elevated YKL-40 levels are associated with the presence [2]–[4] and extent of CAD [2], [27], indicating that YKL-40 could be a quantitative indicator of disease presence and progression, and several studies have documented that YKL-40 levels are elevated in patients suffering from a MI [3]–[5]. It has been discussed previously whether YKL-40 in itself is a pathological participant in the inflammatory process or an opportune and protective response to the actual pathological process. It has been documented that YKL-40 responded to increasing levels of interleukin-1 and tumour necrosis factor-alpha and thereby ihibited the cellular responses to these inflammatory cytokines [1], [28]. This indicates a role of YKL-40 in an ongoing protective process along with the pathological process [28] and could reason our finding, that a doubling in serum YKL-40 or 4th quartile YKL-40 values were independently associated with a 23 (9–35)% respectively 39 (15–56)% decreased risk of first-time IHD. Apparently this seem to be contradictory to previous findings of elevated YKL-40 levels in patients with atrial fibrillation [29], [30], coronary artery disease [2], [3], [5], [31], MI [3], [32] and heart failure [33]. However, these studies documented the actual YKL-40 levels in patients with ongoing CVD but did not investigate the predictive value of YKL-40 in relation to first-time events. YKL-40 may have different roles in the formation of local thrombosis versus the formation of thromboembolisms. Elevated serum YKL-40 levels have been shown in the carotid artery wall in patients with symptomatic (cerebral infarction, transitory cerebral ischemia or infarction of the optical artery) versus asymptomatic atherosclerotic plaques and the highest YKL-40 levels were localized to the lipid-rich core of the atherosclerotic plaque [34]. Moreover, immunohistochemical analyses showed that platelet releasate stimulated the YKL-40 expression by THP-1 monocytes, and that YKL-40 stimulation increased MMP-9-levels in the THP-1 monocytes [34]. These findings suggest YKL-40 to be a marker of plaque instability, potentially reflecting macrophage activation and matrix degradation within the atherosclerotic lesion and support the role of YKL-40 in the formation of thromboembolisms. This could also be the profound explanation for the association between serum YKL-40 levels and the increased risk of ischemic stroke.

Beside the association between rs872129 and the highly elevated mortality risk of ischemic stroke, we could not document that any of the SNPs were associated with a higher prevalence of MI or stroke, with an increased all-cause or cardiovascular mortality or an increased risk of first-time cardiovascular event. As previously stated the found association between rs872129 and risk of fatal ischemic stroke should also be considered with precautions. Rs4950928 has previously been found to contribute to inter-individual variations in YKL-40 levels [13]. The minor G allele of rs4950928 seems to have a negative effect on circulating YKL-40 levels, a finding that is confirmed in the present study, where we documented more than 50% reduced YKL-40 levels in individuals homozygous for this allele. We also documented the same reduction in and influence on YKL-40 levels by the minor T allele of rs10399931 and the minor G allele of rs946263. This is also in accordance with recent studies of patients with CAD (rs10399931) [15], sarcoidosis (10399931) [12] and asthma (rs946263, in perfect LD, r^2^ = 1.0, with rs4960928) [35]. Only rs10399931 and rs4950928 have previously been investigated in relation to cardiovascular disease, but no association has been described [15].

YKL-40 data have not been age-adjusted, since YKL-40 levels increase with age. However, since changes in YKL-40 percentiles in healthy subjects during a 10-year period are small, major increases above an individual's personal age-adjusted percentile might indicate an increased risk of disease with age and not a physiological increase in YKL-40 [36]. In the present study, we cannot argue that an age-adjustment of YKL-40 values would result in significant associations between YKL-40 levels and risk of outcomes. It is a limitation that no differentiation between ischemic versus hemorrhagic stroke during follow-up has been made. A differentiation would most likely have shown, that the association between YKL-40 and the risk of first-time stroke was driven by a very strong association between YKL-40 and risk of ischemic stroke and possible no association with hemorrhagic stroke. Moreover, central registers do not entirely reflect first-time incidences of IHD, since only symptoms that require admission to a hospital are registered. However, this tends to underestimate our findings. Finally, it can be argued that our finding is a chance finding since the large number of analyses increases the risk of a chance finding as well as it can be argued that the study only describes tendencies of *CHI3L1* since the study is of insufficient power to reliably assess associations between SNPs and disease endpoints.

In summary, in this study of 2656 mid-aged and elderly Danes, high YKL-40 levels were predictive of an increased mortality risk of ischemic stroke. Minor allele homozygosity of rs872129 seemed to have a more than 9 times increased risk of fatal ischemic stroke and this genotype was found in approximately 14% of individuals suffering a fatal ischemic stroke. However, final conclusions can not be made of rs872129, since only 0.5% of the population was minor allele homozygotes. YKL-40 levels were also predictive of first-time incidence of stroke during follow-up and of increased all-cause mortality. A decreased risk of IHD with increasing YKL-40 levels was also reported. Beside rs872129 none of the 12 SNPs showed any association with either prevalence of MI or stroke or with risk of primary or secondary outcomes. The results elucidate possible different roles of YKL-40 in the formation of thromboembolisms versus development of local thrombosis, and support the establishment of YKL-40 as a player in the pathogenesis of cerebrovascular disease. The findings are relevant for clinicians and scientists within this field although the clinical implications are sparse for the time being.

## Supporting Information

Table S1 **Prevalence and odds ratio (95% confidence interval) of myocardial infarction and stroke at baseline according to single nucleotide polymorphisms (SNPs) of** ***CHI3L1*** **.**
(DOCX)

Table S2 **Prevalence of all-cause mortality and mortality from ischemic heart disease, ischemic and hemorrhagic stroke and heart failure and of first time incidence of myocardial infarction, angina pectoris/ischemic heart disease and stroke during follow-up according to YKL-40 quartiles at baseline.**
(DOCX)
