# Supplementary material for: Variations of CHI3L1, Levels of the Encoded Glycoprotein YKL-40 and Prediction of Fatal and Non-fatal Ischemic Stroke
Source: PLoS One. 2012 Aug 24;7(8):e43498. doi: 10.1371/journal.pone.0043498 (PMC3427346; doi:10.1371/journal.pone.0043498)
Supplement: Table S1 — Prevalence and odds ratio (95% confidence interval) of myocardial infarction and stroke at baseline according to single nucleotide polymorphisms (SNPs) of CHI3L1 . (DOCX) [file pone.0043498.s001.docx]

Table S1. Prevalence and odds ratio (95% confidence interval) of myocardial infarction and stroke at baseline according to single nucleotide polymorphisms (SNPs) of *CHI3L1*.

| SNP | Genotype | Myocardial infarction | | Stroke | |
| --- | --- | --- | --- | --- | --- |
|  |  | Prevalence | OR (95% CI)‡ | Prevalence | OR (95% CI)‡ |
| rs10399931 | CC* | 2.5 (37/1476)** | 1 | 1.4 (20/1475)** | 1 |
|  | CT | 3.5 (33/933) | 1.46 (0.90; 2.37) | 0.8 (7/933) | 0.55 (0.23; 1.30) |
|  | TT | 2.8 (4/141) | 1.08 (0.37; 3.14) | 0.7 (1/141) | 0.49 (0.07; 3.69) |
|  |  | p=0.28† | p=0.32 | p=0.72† | p=0.31 |
| rs12123883 | TT* | 3.3 (71/2165) | 1 | 1.1 (24/2163) | 1 |
|  | TC | 1.5 (6/389) | 0.50 (0.21; 1.18) | 0.8 (3/390) | 0.73 (0.22; 2.43) |
|  | CC | 0.0 (0/13) | 0 | 7.7 (1/13) | 6.72 (0.81; 56.05) |
|  |  | p=0.23 | p=0.11 | p=0.57 | p=0.31 |
| rs2486064 | GG* | 3.3 (27/811) | 1 | 1.4 (11/810) | 1 |
|  | GA | 2.4 (30/1246) | 0.71 (0.41; 1.22) | 1.0 (12/1246) | 0.71 (0.31; 1.62) |
|  | AA | 4.1 (20/489) | 1.17 (0.64; 2.14) | 1.0 (5/489) | 0.72 (0.25; 2.10) |
|  |  | p=0.22 | p=0.21 | p=0.84 | p=0.70 |
| rs2886117 | GG* | 2.8 (55/1942) | 1 | 0.9 (17/1941) | 1 |
|  | GA | 3.0 (16/542) | 1.04 (0.58; 1.85) | 1.7 (9/542) | 1.92 (0.85; 4.33) |
|  | AA | 4.9 (2/41) | 1.90 (0.43; 8.44) | 2.4 (1/41) | 2.58 (0.33; 20.08) |
|  |  | p=0.47 | p=0.74 | p=0.53 | p=0.26 |
| rs4950928 | CC* | 2.6 (42/1590) | 1 | 1.4 (22/1589) | 1 |
|  | CG | 3.4 (29/854) | 1.32 (0.81; 2.16) | 0.6 (5/854) | 0.41 (1.6; 1.10) |
|  | GG | 3.5 (4/113) | 1.20 (0.41; 3.47) | 0.9 (1/113) | 0.57 (0.08; 4.28) |
|  |  | p=0.46 | p=0.55 | p=0.39 | p=0.15 |
| rs4950930 | GG* | 2.9 (66/2317) | 1 | 0.9 (22/2317) | 1 |
|  | GA | 4.0 (9/225) | 1.25 (0.60; 2.60) | 2.7 (6/224) | 2.8 (1.2; 7.0) |
|  | AA | 14.3 (1/7) | 9.03 (0.98; 82.95) | 0.0 (0/7) | 0 |
|  |  | p=0.60 | p=0.26 | p=0.29 | p=0.13 |
| rs6691378 | GG* | 3.0 (59/1999) | 1 | 0.9 (18/1998) | 1 |
|  | GA | 3.1 (16/518) | 1.06 (0.60; 1.88) | 1.9 (10/518) | 2.17 (0.99; 4.74) |
|  | AA | 2.6 (1/39) | 1.06 (0.14;8.11) | 0.0 (0/33) | 0 |
|  |  | p=0.99 | p=0.98 | p=0.31 | p=0.12 |
| rs871799 | GG* | 2.9 (60/2074) | 1 | 1.0 (21/2074) | 1 |
|  | GC | 3.8 (17/448) | 1.33 (0.76; 2.33) | 1.6 (7/447) | 1.51 (0.64; 3.59) |
|  | CC | 0.0 (0/32) | 0 | 0.0 (0/32) | 0 |
|  |  | p=0.34 | p=0.22 | p=0.70 | p=0.46 |
| rs872129 | AA* | 3.2 (68/2158) | 1 | 0.9 (20/2158) | 1 |
|  | AG | 2.3 (9/386) | 0.75 (0.37; 1.54) | 1.8 (7/385) | 2.01 (0.84; 4.80) |
|  | GG | 0.0 (0/12) | 0 | 0.0 (0/12) | 0 |
|  |  | p=0.56 | p=0.49 | p=0.42 | p=0.29 |
| rs880633 | CC* | 2.6 (19/733) | 1 | 1.0 (7/732) | 1 |
|  | CT | 3.0 (39/1283) | 1.13 (0.64; 2.00) | 1.1 (14/1283) | 1.10 (0.44; 2.76) |
|  | TT | 3.4 (18/536) | 1.28 (0.66; 2.50) | 1.3 (7/536) | 1.33 (0.46; 3.81) |
|  |  | p=0.96 | p=0.77 | p=0.97 | p=0.87 |
| rs883125 | CC* | 3.2 (58/1841) | 1 | 0.9 (16/1839) | 1 |
|  | CG | 2.7 (18/672) | 0.79 (0.45; 1.36) | 1.8 (12/673) | 2.04 (0.96; 4.36) |
|  | GG | 0.0 (0/56) | 0 | 0.0 (0/56) | 0 |
|  |  | p=0.25 | p=0.16 | p=0.31 | p=0.10 |
| rs946263 | AA* | 2.6 (43/1645) | 1 | 1.4 (23/1644) | 1 |
|  | AG | 3.7 (30/812) | 1.45 (0.89; 2.35) | 0.5 (4/812) | 0.34 (0.12; 0.99) |
|  | GG | 4.3 (4/92) | 1.38 (0.47; 4.03) | 1.1 (1/92) | 0.65 (0.09; 4.9) |
|  |  | p=0.41 | p=0.32 | p=0.27 | p=0.09 |

* Major allele

** % (n/ n_total_). N_total_ may differ due to missing data.

† p values of chi square test.

‡ OR (95% CI) were estimated in logistic regression models. Models were adjusted for age and gender. p values of likelihood ratio test
