# Supplementary material for: Variations of CHI3L1, Levels of the Encoded Glycoprotein YKL-40 and Prediction of Fatal and Non-fatal Ischemic Stroke
Source: PLoS One. 2012 Aug 24;7(8):e43498. doi: 10.1371/journal.pone.0043498 (PMC3427346; doi:10.1371/journal.pone.0043498)
Supplement: Table S2 — Prevalence of all-cause mortality and mortality from ischemic heart disease, ischemic and hemorrhagic stroke and heart failure and of first time incidence of myocardial infarction, angina pectoris/ischemic heart disease and stroke during follow-up according to YKL-40 quartiles at baseline. (DOCX) [file pone.0043498.s002.docx]

Table S2. Prevalence of all-cause mortality and mortality from ischemic heart disease, ischemic and hemorrhagic stroke and heart failure and of first time incidence of myocardial infarction, angina pectoris/ischemic heart disease and stroke during follow-up according to YKL-40 quartiles at baseline.

|  | 1^st^ quartile | 2^nd^ quartile | 3^rd^ quartile | 4^th^ quartile | p value |
| --- | --- | --- | --- | --- | --- |
| YKL-40, range, ng/ml | ≤ 40 | > 40, ≤ 57 | > 57, ≤ 85 | > 85 |  |
| Primary endpoints, mortality: |  |  |  |  |  |
| All-causes | 55 (8.1) | 79 (11.8) | 122 (19.3) | 214 (32.4) | <0.0001 |
| Ischemic heart disease | 13 (1.9) | 9 (1.3) | 21 (3.3) | 36 (5.5) | <0.0001 |
| Ischemic stroke | 1 (0.001) | 3 (0.04) | 5 (0.08) | 13 (2.0) | 0.001 |
| Hemorrhagic stroke | 0 (0.0) | 5 (0.7) | 9 (1.4) | 4 (0.6) | 0.02 |
| Heart failure | 1 (0.01) | 1 (0.01) | 3 (0.5) | 9 (1.4) | 0.002 |
| Secondary endpoints: |  |  |  |  |  |
| Myocardial infarction | 24 (3.5) | 23 (3.4) | 27 (4.3) | 33 (5.0) | <0.0001 |
| Ischemic heart disease | 50 (7.4) | 59 (8.8) | 65 (10.3) | 57 (8.6) | 0.33 |
| Stroke | 30 (4.4) | 49 (7.3) | 51 (8.1) | 96 (14.5) | <0.0001 |

Presented as N (% within YKL-40 quartile)
